# The use of an individualized intraoperative video shows no impact on the early postoperative clinical outcome after total knee arthroplasty: a prospective, randomized, controlled trial

**DOI:** 10.1007/s00402-022-04755-0

**Published:** 2023-01-04

**Authors:** Patrick Sadoghi, Christoph Listl, Jan Lewis, Patrick Reinbacher, Andreas Leithner, Georg Hauer

**Affiliations:** Department of Orthopaedics and Trauma, Medical University of Graz, Auenbruggerplatz 5, 8036 Graz, Austria

**Keywords:** Total knee arthroplasty, Total knee replacement, Video demonstration, Recovery, Fast-track

## Abstract

**Purpose:**

The purpose of the study was to evaluate the potential of an intraoperatively recorded video shown to patients immediately postoperatively on early outcome after total knee arthroplasty (TKA). The hypothesis was that there is a beneficial outcome concerning range of motion (ROM) and patient-reported outcome due to enhanced trust into the artificial joint.

**Methods:**

Seventy-three patients were randomly assigned 1:1 to two study groups in which they were either shown a video of their own postoperative range of motion or they were not. Clinically, the New Knee Society Score (nKSS) and ROM were evaluated and compared between the groups 6 weeks after surgery. Chi-square exact test, Kolmogorov–Smirnov test, Mann–Whitney *U* test, and the Wilcoxon signed rank test were used. Inter- and intra-class correlations were calculated for measurements of ROM.

**Results:**

No clinically relevant differences were observed preoperatively and 6 weeks postoperatively between both groups in range of motion (ROM). All patients were showing a significantly improved clinical outcome 6 weeks after the procedure. Clinical scores showed statistically significant differences with respect to preoperative nKSS for satisfaction and statistically significant differences with respect to postoperative nKSS for function.

**Conclusion:**

Showing a video filmed immediately after implantation of primary TKA had no significant effect on ROM and clinical outcome at 6 weeks. We believe that face-to-face verbal communication in combination with video-assisted education ensures that patients understand their artificial joint in the best possible way and will continue to use intraoperatively filmed videos to enhance patient engagement during postoperative rehabilitation.

**Level of evidence:**

I.

## Introduction

It is an ongoing challenge to continuously improve the outcome after total knee arthroplasty (TKA). Although TKA is considered a successful and cost-effective surgical procedure, every fifth patient reports dissatisfaction despite improvements in the surgical technique and implants [1–3]. Identification of the causes of dissatisfaction is important and a lack of postoperative range of motion (ROM) and missing trust in the artificial joint itself can contribute to unsatisfied patients.

Evidence suggests that rapid early mobilization is critical for patients to regain functional knee ROM [4, 5]. Without early intentional effort, the final result may show a lack of ROM, and TKA is not achieving its goal of relieving pain and restoring function in a substantial proportion of patients [4]. Many studies have reported that patient education optimizes patient engagement, satisfaction, and functional outcome [5–8]. Furthermore, poor patient motivation, immobility, and a delay in starting a rehabilitation program can contribute to a loss of ROM and painful stiffness known as arthrofibrosis [9].

Various methods have been described for patient education including group classes, booklets, and videotapes [10, 11]. To date, however, no study has investigated the benefit of an intraoperatively recorded video shown to patients immediately postoperatively, where they are shown the full extent of ROM of their own artificial knee joint. The aim of this study was to evaluate its potential on early outcome after TKA. The hypothesis of the study was that this video illustration would increase confidence in the operated knee and allow patients to perform better in the immediate postoperative phase with respect to ROM and clinical outcome parameters.

## Methods

### Study design

**Fig. 1:**
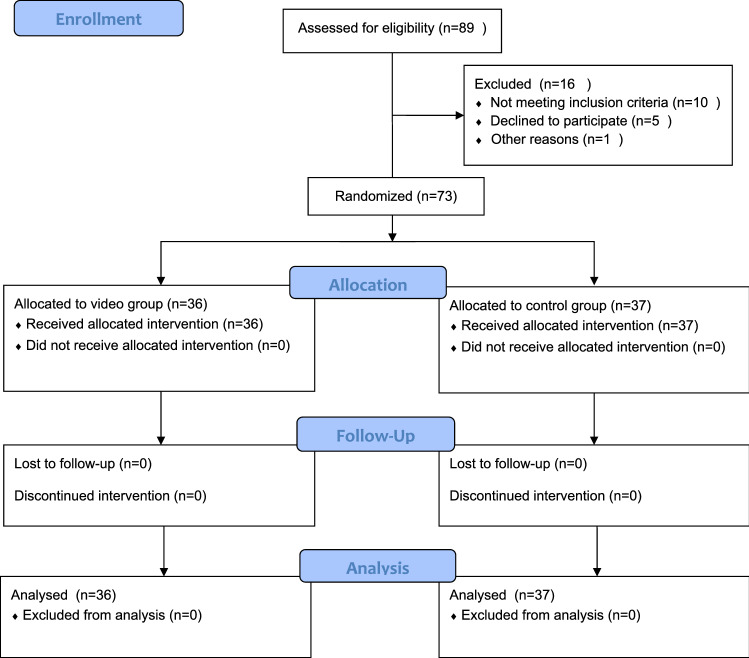
CONSORT flow diagram showing patient enrollment, randomization, and analysis CONSORT, Consolidated Standards of Reporting Trials

This is a prospective, randomized, controlled, parallel group trial conducted at a single high-volume orthopedic surgery center with an equal allocation rate. Eligible participants were all patients undergoing primary elective TKA under the care of one highly experienced senior knee surgeon within 8 months. Written informed consent was obtained from all patients before any study procedures were conducted. A total of 89 consecutive patients were screened for potential eligibility to participate in this study (Fig. 1).

### Study population

Patients eligible for randomization were suffering from severe primary osteoarthritis of the knee confirmed by weight-bearing anteroposterior and lateral radiographs (Kellgren–Lawrence Score III/IV). Exclusion criteria were valgus deformity > 20° (*n* = 2), rheumatoid arthritis (*n* = 1), BMI > 35 (*n* = 5), patients with known or suspected metal allergies (*n* = 2), and patients with active or recently treated infections (*n* = 2). Patients who were unwilling to participate (*n* = 5) or unable to provide informed consent (*n* = 1) were also excluded.

Of the 89 subjects screened, 73 patients were included in the study. No differences in preoperative demographic data with respect to age, gender, body mass index, and ASA score were observed between both groups (Table 1).

**Table 1:** Differences in demographic variables between groups

	Control group	Video group	*p* value
Age at surgery (in years)	72.2 ± 8.7	69.0 ± 10.0	0.154^a^
Gender			
Male	12 (33.3%)	12 (32.4%)	0.935^b^
Female	24 (66.7%)	25 (67.6%)	
BMI	30.7 ± 5.9	30.2 ± 6.5	0.743^a^
ASA			
1	1 (2.8%)	3 (8.1%)	0.386^b^
2	10 (27.8%)	11 (29.7%)	
3	23 (63.9%)	23 (62.2%)	
4	2 (5.6%)	0 (0.0%)	

^a^*t* test

^b^Chi-squared test

Subjects were randomly assigned 1:1 to two study groups in which they were either shown a video of their own postoperative range of motion or they were not. Randomization was performed using the Randomizer for Clinical Trials tool developed at the Medical University of Graz. The participants were not informed of their group allocation and were not aware of the study’s hypothesis. There were no significant differences in the demographic data between the two groups (Table 1).

### Postoperative video

**Fig. 2:**
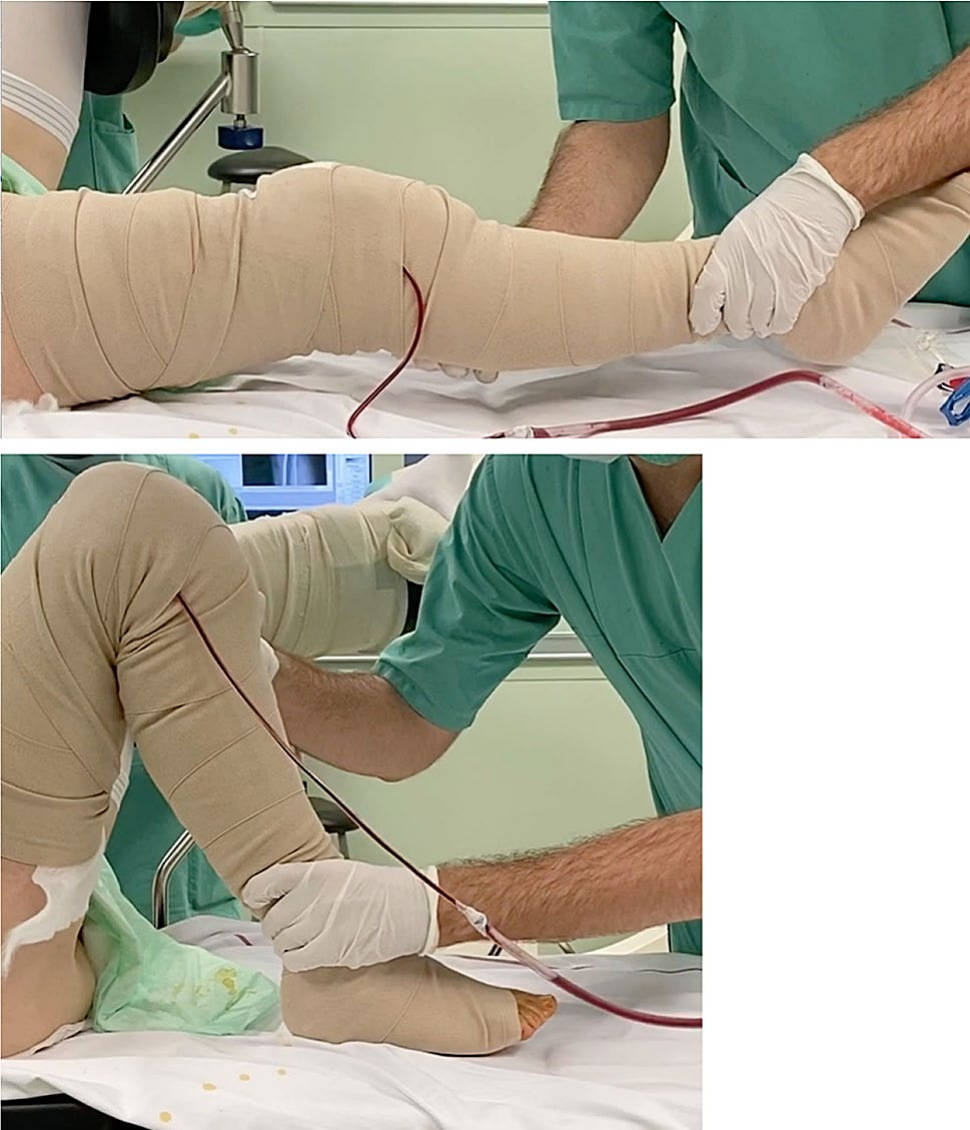
Parts of the video shown to the patients demonstrating the full ROM of the artificial joint

On the morning of the first postoperative day, the study participants from the video group were shown a 4 K video, which was recorded directly postoperatively. The video lasted 60 s and showed the repeated passive extension and flexion movement of the patient's own operated knee joint while still on the operating table with full extension and flexion of 125° (Fig. 2). Before the video was started, a unique assignment of the video was ensured by the name tag. There was no doubt about the authenticity of the video. The artificial knee joint was moved by the surgeon. The video was recorded with a standard mobile phone camera immediately after the skin was sutured and the dressing applied. When the video was shown, it was explained to the patients that according to the video the prosthesis design and the postoperative result allowed complete ROM.

### Surgical procedure

All patients received cemented (Palacos R + G, Heraeus Medical, Wehrheim, Germany), fixed-bearing, cruciate-retaining Attune (DePuy Synthes, Warsaw, IN) TKA without patella resurfacing. Participants underwent the same standardized preoperative protocol and surgical technique. All surgeries were performed via the medial parapatellar approach and the tibia first method with the balancing of the flexion gap. After TKA surgery, all subjects received a standardized rehabilitation protocol under the supervision of a physiotherapist at the hospital. The protocol included full weight bearing immediately after surgery with the use of crutches, continuous passive motion (CPM) therapy, active ROM and strengthening exercises, and gait training. A standardized home-based exercise program was followed.

### Outcome measures

All patients were evaluated the day before surgery and were then re-evaluated 6 weeks postoperatively using the German version of the 2011 New Knee Society Score (nKSS). For the preoperative and 6 weeks postoperative nKSS, the patients were asked to grade their symptoms (maximum of 25 points), satisfaction (maximum of 40 points), expectations (maximum of 15 points), and functional activities (maximum of 100 points). The ROM was measured with a double-armed goniometer twice by two observers.

The study procedure followed accepted ethical, scientific, and medical standards and was conducted in compliance with recognized international standards, including the principles of the Declaration of Helsinki. Informed consent was obtained from all the study participants and the study protocol was approved by the local ethics committee (31-219 ex 18/19).

### Statistical analysis

Data were analyzed by SPSS Version 26.0 (IBM Corporation, New York, USA). Descriptive statistics for continuous variables were reported as the mean and standard deviation (SD). Categorical variables were reported as count and proportions. For comparisons of categorical variables, the Chi-square exact test was used. Normality of distribution was assessed using the Kolmogorov–Smirnov test, which revealed a non-parametric distribution for all variables of interest. Differences between preoperative and postoperative data were observed through the Mann–Whitney *U* test and the Wilcoxon signed rank test. The significance level was set at *p* < 0.05. An a priori power analysis revealed that a minimum of 36 patients per group would be necessary to detect a clinically relevant difference of 10° in ROM with a SD of 25° at a significance level of *p* < 0.05 with a power greater than 0.8. Inter- and intra-class correlations were calculated for measurements of range of motion. The magnitude of the difference between both groups reached a post hoc power greater than 80% (according to Hoenig and Heisey).

## Results

With respect to the main endpoint range of motion (ROM), no clinically relevant differences were observed preoperatively (*p* = 0.388) and 6 weeks postoperatively between (*p* = 0.423) both groups (Table 2), and all patients were showing a significantly improved clinical outcome 6 weeks after (*p* = 0.022) the procedure (Table 2). Clinical scores showed statistically significant differences with respect to preoperative nKSS (*p* = 0.008) for satisfaction and statistically significant differences with respect to postoperative nKSS (*p* = 0.012) for function, both without clinical relevance (Tables 3, 4). Inter- and intra-class correlations revealed substantial agreement greater than 80% each, for measurements of range of motion. We report no patient lost to follow-up at final evaluation.

**Table 2:** Comparison of range of motion and functional activities before surgery and 6 weeks postoperatively in both groups

	Control group	Video group	*p* value^a^
Pre mean knee flexion ° (SD)	106.0 (13.4)	103.4 (12.1)	0.388
6 w Mean knee flexion, ° (SD)	99.4 (9.8)	101.2 (9.0)	0.423
*p* value^b^	**0.008**	0.384	
Pre mean nKSS function	30.6 (13.7)	36.7 (16.0)	0.085
6 w Mean nKSS function	38.3 (15.0)	46.8 (15.7)	**0.022**
*p* value^b^	**0.018**	**0.003**	

All statistically significant differences are highlighted in bold font

*SD* standard deviation

^a^Unpaired *t* test

^b^Paired *t* test

**Table 3:** Preoperative clinical scores as medians with interquartile ranges (IQR)

Preoperative clinical score	Control group	Video group	*p* value^a^
Pre nKSS symptoms	11 (7–13)	12 (10–14)	0.109
Pre nKSS satisfaction	10 (6–12)	8 (12–16)	**0.008**
Pre nKSS expectations	13 (12–14)	13 (12–14)	0.564
Pre nKSS functional activities	29 (21–39)	32 (24–49)	0.135

All statistically significant differences are highlighted in bold font

*nKSS* new knee society score, *IQR* interquartile range

^a^Wilcoxon signed rank test

**Table 4:** 6-week clinical scores as medians with interquartile ranges (IQR)

6 w Clinical score	Control group	Video group	*p* value^a^
6 w nKSS symptoms	21 (19–23)	21 (19–25)	0.382
6 w nKSS satisfaction	30 (27–35)	30 (27–34)	0.874
6 w nKSS expectations	11 (9–12)	9 (9–12)	0.300
6 w nKSS functional activities	35 (27–46)	49 (38–57)	**0.012**

All statistically significant differences are highlighted in bold font

*6 w* 6 weeks, *nKSS* new knee society score, *IQR* interquartile range

^a^Wilcoxon signed rank test

## Discussion

The aim of this study was to evaluate the intraoperatively recorded video’s potential on the early outcome after TKA. The hypothesis was that this intraoperative video material would increase confidence in the operated knee and allow patients to perform better in the immediate postoperative phase with respect to the ROM and clinical outcome parameters. We found that the showing of an intraoperatively recorded video illustrating ROM had no effect on ROM and clinical outcome at 6 weeks and that both groups showed significant improvements in clinical scores.

The theoretical concept of the study design was “seeing is believing”. The study aimed to improve the postoperative ROM and clinical scores by showing patients their actual potential of knee movement and stability. There are no other studies that look at postoperative education and its effect on outcome like this one. The participants who have been shown a video should be encouraged to participate more actively and enthusiastically immediately postoperatively. The patients should be given a feeling of safety and trust in the prosthesis as anxiety is a known negative predictor of unsatisfactory results after joint replacement [12–14]. Psychological factors play a key role in recovery and increased patient activation is known to be beneficial and improves patient satisfaction and patient-reported outcomes [15]. According to the current findings, however, the patients’ ability and willingness in the immediate postoperative course could not be improved. The visual illustration alone had no positive impact on ROM and clinical outcome after TKA at a short-term follow-up. Nevertheless, digital educational tools are now an established part of patient care. Studies have already proven that the digital approach can be more effective than traditional booklets [10, 11]. Additionally, it has been recommended that an advanced postoperative training is more effective to quickly get back into daily life [16, 17].

In TKA patients, recovery starts immediately after surgery. Besides preoperative education, postoperative care is crucial for achieving a favorable outcome [18–20]. In our experience, a considerable proportion of patients lack understanding how they are allowed to bear weight after surgery, what the operated joint can tolerate, and what activities are allowed. With a postoperative video illustration, people might be less anxious and are more courageous in exercises.

The follow-up period was limited to 6 weeks to minimize other factors that influence the outcome, although we recognize that improvement of TKA may extend well beyond this period. The first 6 weeks are relatively comparable for all patients. Usually, our patients start with outpatient post-surgical rehabilitation on the day of discharge. After 6–10 weeks, some patients go to inpatient rehabilitation, and some continue with outpatient physiotherapy. The rest is already so satisfied or independent after 6 weeks that no further professional therapy is needed. Before the study began, we did not expect a measurable or clinically relevant effect of the video beyond 6 weeks.

Surprisingly, after 6 weeks, the ROM had not yet reached the preoperative status. This may be related to the fact that due to the corona pandemic restrictions, the nKSS of six patients from the video group and five patients from the control group had to be assessed by telephone. It is possible that these patients underreported their ROM, although TKA patients are usually very familiar with the degrees of flexion, as they are monitored by this method from the first postoperative day. The outpatient follow-up of these 11 patients could only take place later.

We want to outline the following limitation of our work: The clinical outcome and ROM as the endpoints of this analysis were evaluated only within a short follow-up period of 6 weeks and long-term data cannot be presented at this point. In addition, it might have been an option to collect data on pain medication, length of hospital stay, and other endpoints to detect potential short-term benefits in the control group. Normally, inter- and intra-class calculations are performed with a break of 2 weeks in between, which was not possible due to the chance of ROM improvement within this time period. However, we also want to emphasize the benefit that this is a randomized, controlled, prospective trial with an a priori power analysis and sample size calculation. The fact that we observed no clinically relevant differences between groups does not impair the scientific methodology, as we performed an adequate power analysis, but further strengthens the relevance of the publication of these results due to a potential file-drawer bias if negative outcome studies are not published [21].

## Conclusion

Showing a video filmed immediately after implantation of primary TKA had no significant positive effect on ROM and clinical outcome at 6 weeks. We believe that face-to-face verbal communication in combination with video-assisted illustrations ensures that patients understand their artificial joint in the best possible way and will continue to use intraoperatively filmed videos to enhance patient engagement during postoperative rehabilitation.
